# The Effect of Lifestyle Intervention on Pregnancy and Birth Outcomes on Obese Infertile Women: A Systematic Review and Meta-Analysis

**DOI:** 10.22074/ijfs.2020.5921

**Published:** 2020-02-25

**Authors:** Juan J Espinós, Ivan Solà, Claudia Valli, Ana Polo, Lucja Ziolkowska, M José Martínez-Zapata

**Affiliations:** 1 Department of Obstetrics and Gynaecology, Hospital de la Santa Creu i Sant Pau, Barcelona, Universitat Autònoma de Barcelona (UAB), Bellaterra, Spain; 2 IbCC Iberoamerican Cochrane Center, Barcelona, Spain; 3 CIBERESP CIBER Epidemiología y Salud Pública (CIBERESP), Barcelona, Spain; 4 IIB Sant Pau Biomedical Research Institute Sant Pau (IIB Sant Pau), Barcelona, Spain; 5 Department of Reproduction Puigvert Foundation, Barcelona, Spain; 6 Medical University of Silesia, Katowice, Poland

**Keywords:** Diet, Infertility, Live Birth Rate, Obesity, Physical Exercise

## Abstract

Obesity has been associated with negative effects on natural fertility and poor prognosis when assisted reproductive technologies (ART) are performed. Patients attending for fertility treatments are often advised to optimize their weights to improve the outcomes. There is lack of enough information on how weight-loss would be effective for improving fertility in women who are overweight or obese. We conducted a systematic review to evaluate whether weight-loss achieved by lifestyle program improves natural or assisted reproduction in obese infertile women. We searched CENTRAL, MEDLINE, and EMBASE up to March 2018. Two reviews were selected as randomised trials assessing a lifestyle intervention in women with obesity before receiving treatments for infertility and appraised their risk of bias. We extracted data on pregnancy, birth, and miscarriage rates as the primary outcomes and pooled effect estimates using a random effects model. The primary outcome was the live birth rate. We reported summary measures as the relative risk (RR), 95% confidence interval (CI), and percentage of heterogeneity (I2). We included eight randomised trials with 1175 women. Lifestyle programmes, improved pregnancy rates (RR: 1.43, CI: 95% 1.02 to 2.01; I2=60%; 8 RCTs; N=1098) but had no impact on live births (RR: 1.39, CI: 95% 0.90 to 2.14; I2=64%; 7RCTs; N=1034). Our findings suggest that women participating in lifestyle interventions had an increased risk of miscarriage (RR: 1.50, CI: 95% 1.04 to 2.16; I2=0; 6RCTs; N=543). We rated the quality of evidence for these outcomes as the moderate-to-low. Lifestyle interventions slightly increased the pregnancy rate, while it would be uncertain whether it can improve the live birth. Lifestyle interventions can increase the risk of miscarriage. More research is needed to further explore lifestyle interventions on reproductive outcomes in obese infertile women.

## Introduction

The prevalence of overweight and obesity among women have increased more than three times in the last years, creating a global pandemic affecting both industrialized and developing countries (1, 2). Obesity has been associated with negative effects on both general and reproductive health. Natural fertility is compromised in both, men and women (3). In the last, polycystic ovarian disease (which is typically associated with central obesity, insulin resistance, and hyperinsulinism) and alterations affecting obesity-related hormones (e.g., leptin, adipokines, ghrelin, and endorphins) can affect oocyte quality, fertilization, embryo development, and implantation, as well as reducing the fertility rate in women with a normal menstrual cycle (4-7). The extent of impact of obesity on *in vitro* fertilization (IVF) outcomes is unknown due to the heterogeneity of studies conducted in this area, the retrospective nature of most investigations, and lack of standardized criteria (8-10). Obesity has been associated with an increase in gonadotropin need, more days of treatment, higher cancellation rates of cycles due to the inadequate response, decreased numbers of total and mature eggs, reduced rates of fertilization, and consequently fewer high-quality embryos. Obesity has also been associated with endometrial abnormalities and lower implantation rates (11-14). 

Weight-loss has been appreciated as one of the most effective means of increasing the probability of fertility in infertile overweight or obese women (15, 16). Few studies have analyzed the actual effects of a lifestyle intervention, including diet and exercise on obese women wishing to become pregnant. Additionally, the findings of these studies have been inconsistent, probably owing to methodological shortcomings (17). A prior systematic review, including randomized and non-randomized controlled trials and studies using weight reduction drugs showed an increase in the feasibility of becoming pregnant, with no significant adverse effect on live birth rates (18).

In this systematic review, we aimed to evaluate whether weight-loss achieved by a lifestyle intervention improved the pregnancy outcomes in obese infertile women, with a specific focus on the live birth rate. 

## Materials and Methods

We conducted this systematic review according to the methodological guidance of Cochrane (19). We reported the findings from the review according the PRISMA statement (20).

### Search strategies

We searched MEDLINE (via PubMed), EMBASE (via Ovid), and CENTRAL (via The Cochrane Library) from the databases inception up to March 2018. We designed a search strategy combining text words and controlled vocabulary adapted to the requirements of each database. We included the complete search strings in the Materials S1 (See Supplementary Online Information at www.ijfs.ir). Additionally, we searched the reference list of all eligible studies and contacted authors of the included trials to request additional information.

### Study selection

We included randomised controlled trials assessing a lifestyle intervention in obese women before receiving treatments for infertility. The lifestyle interventions that we considered in this study consisted of any type of structured physical exercise and/or any low calorie intake diet refered by the primary included studies. Eligible trials included women with a body mass index of 29 or higher who were candidates for IVF. The selected trials assessed the structured health promotion programmes consisting of dietary intake reduction alone or combined with physical activity compared with an inactive control group (e. g. women on a waiting list) or women receiving weight loss advice. Three authors independently evaluated whether the references retrieved from the searches met the inclusion criteria and resolved disagreements by discussion or through adjudication by an additional author. We obtained full copies of eligible references for a final decision with respective to their inclusion and reported the reason that led to exclusion of studies.

### Outcomes

We set the following primary outcomes: live birth (including spontaneous live birth, IVF live birth and cumulative live birth per initial cycle), cumulative pregnancy rate and miscarriage (pregnancy ending within the first 20 weeks of gestation). Secondary outcomes were pregnancy (including multiple pregnancies), ongoing pregnancy, and implantation rates.

### Data extraction and risk of bias assessment

Two authors extracted independently the relevant data from chosen trials using a predefined extraction form and an additional author revised the process for accuracy. We registered the characteristics of included studies in descriptive tables. We contacted authors from included studies to request missing data in published papers.

We assessed independently the risk of bias from included trials using the Cochrane tool for that purpose (21). We assessed the trial randomisation sequence generation and its concealment, the concealment of the intervention to participants, researchers, and outcomes assessors, attrition, and incomplete outcome data and selective outcome reporting.

### Data analysis and findings description

We analysed the effect measures for dichotomous variables using risk ratios (RR) and mean differences (MD) for continuous variables calculating their 95% confidence intervals (CI). We considered statistic significant difference between compared groups when 95% CI was not included. The unit of the analysis of interest was the participants in included trials and we used the available-case analysis approach to calculate the effect estimates.

When appropriate, we calculated pooled effect estimates for each outcome using a fixed-effect model or a random effect model when there was statistical heterogeneity (22). We assessed heterogeneity comparing characteristics from included studies and through the I square statistics (23) considering a substantial statistical heterogeneity for values greater than 50% and considerable heterogeneity for values greater than 75% scenario in which we did not perform the pool effect estimates. We performed sub-group analyses according to the lifestyle programme assessed in the included trials (diet alone or combined with physical activity). We planned sensitivity analyses excluding trials with the highest risk of bias or those that were a suspected source of heterogeneity. As any pooled analyses included more than 10 trials, we were not able to conduct formal tests to assess the impact of publication bias (24). We used the statistical package in the open access software Review Manager (v 5.3.5) to conduct all of the analyses (25). We assessed the quality of evidence to judge the confidence in the effect estimates obtained from each primary outcome. We rated the quality of evidence as high, moderate, low or very low according to the impact of each outcome on the risk of bias, indirectness, and effect estimates inconsistency, and imprecision (26). We summarized the effect estimates for primary outcomes and their quality of evidence in a summary of the Table of findings (27).

## Results

### Study selection and characteristics

**Fig 1:**
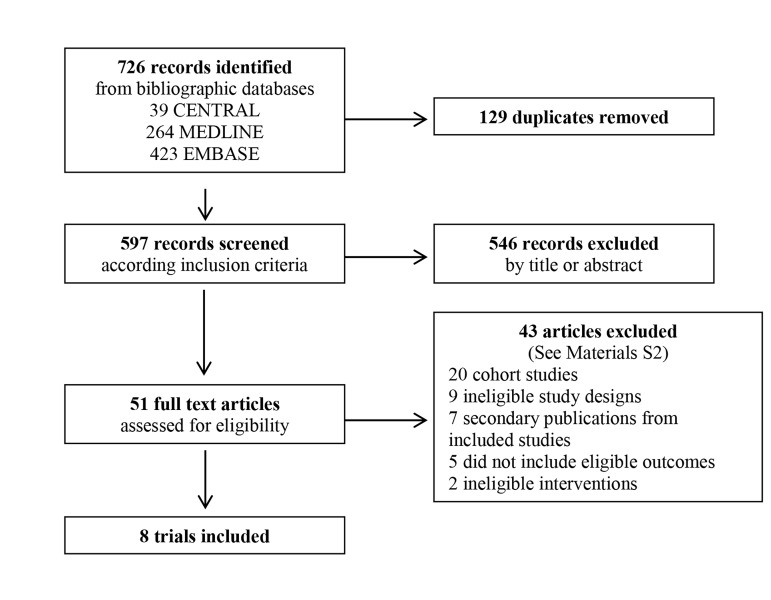
Flowchart for study eligibility.

Our search strategy yielded 726 records of which 48 were potentially eligible to be included. The flowchart (Fig .1) describes the complete eligibility process, and we describe the reasons for excluding 40 studies and the main characteristics of eight included trials (28-34) in the Materials S2 (See Supplementary Online Information at www.ijfs.ir) and the Table 1, respectively. Table 2 shows the summary of findings of the review with a judgement on their quality of evidence.

In total, we included 1175 infertile women. The mean age ranged from 29 to 34 years old, and the body mass index (BMI) from 24 to 38. The included trials compared lifestyle-structured programmes with the usual care. The assessed programmes consisted of dietary intake reduction (28-30) or combined with physical activity interventions (6, 30-33). Women in control groups immediately received infertility treatment with no history of interventions or were included in a waiting list for IVF (28-30, 32) or received standard advice for weight-loss (16, 31, 34). All lifestyle interventions significantly reduced the weight of infertile women compared with control group in the Materials S3 (See Supplementary Online Information at www.ijfs.ir). The mean weight loos values ranged between 3 and 10 kg at the end of the intervention. We did not pooled the results of studies reporting weight-loss due to the presence of high heterogeneity (95%).

### Risk of bias

**Fig 2:**
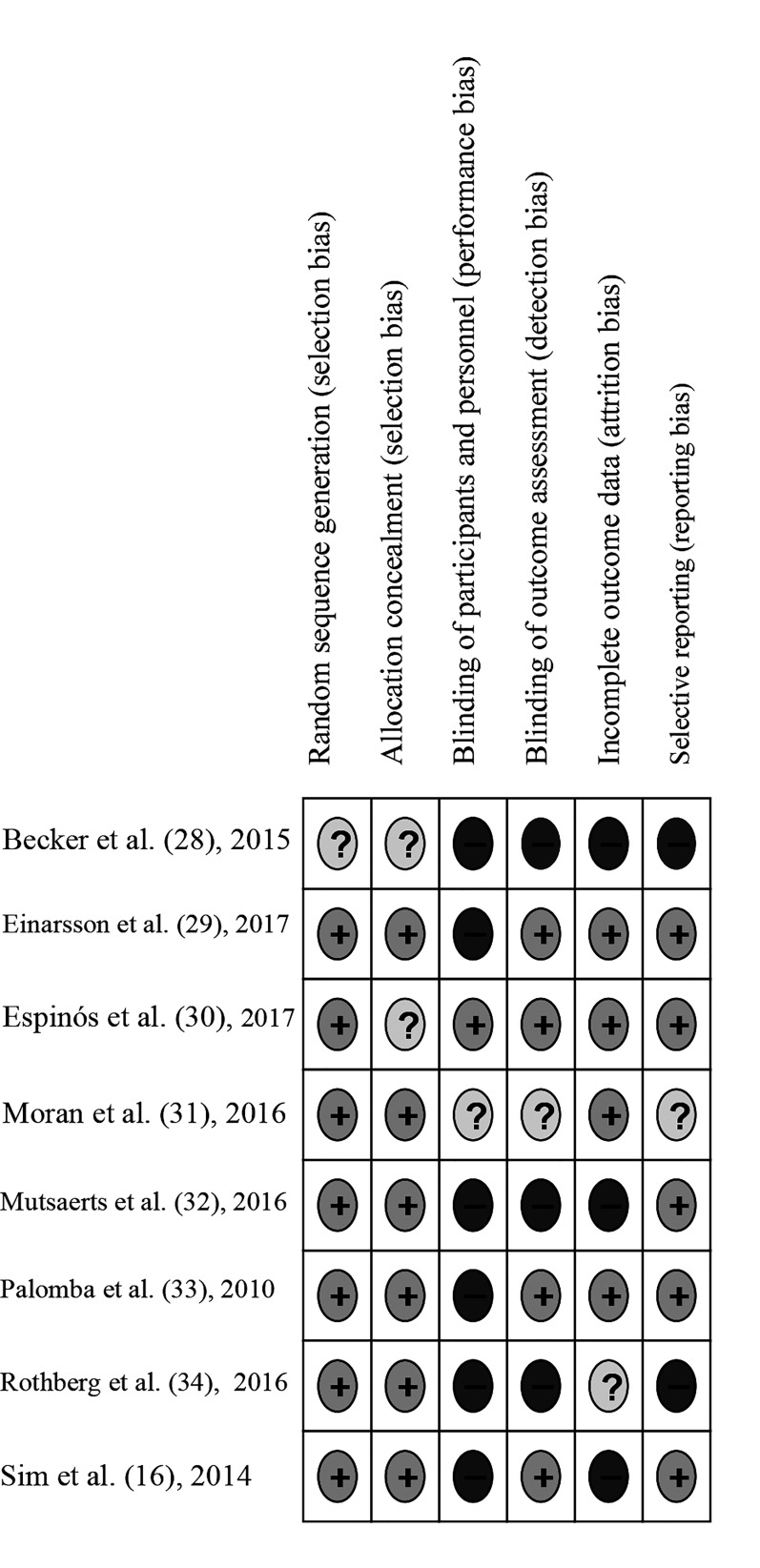
Risk of bias.

Most trials implemented random sequences generated adequately using lists of computer generated numbers (16, 29-34) and had proper allocation concealment, using opaque envelopes in most of the cases (16, 31-34). With the exception of one trial (30), the rest was open or did not provide details on blinding of researchers or participants, but four implemented a blinded outcome assessment (29- 32). We considered three trials having high risk of bias because the data available for the analysis were partially complete (16, 28, 32). Finally, two trials had high risk of selective reporting bias because some outcomes included in their protocols did not coincide with those reported in the published reports of their findings (Fig .2) (28, 34).

**Table 1:** Characteristics of included studies


Study ID, Setting, country	Women	Age (Y) Mean years (SD) Experimental/control group	BMI at baseline Mean (SD) Experimental/control group	Experimental intervention	Control intervention	Outcomes	Follow-up (months)	Funding

Becker et al. (28), 2015 Obstetrics and Gynaecology Service of the Hospital de Clinicas de Porto Alegre, Brazil	35	31.36 (SE 0.89)/31.25 (SE 0.78)	28.67 (SE 0.60)/ 28.82 (SE 0.98)	Hypocaloric diet with a low glycemic index and low glycemic load	Maintenance of the body weights and usual diets	Live birth (spontaneous) ndesirable effects (miscarriage) Pregnancy rate (clinical) BMI change Weight change	12	Not reported
Einarsson et al. (29), 2017 Infertility clinics Sweden, Denmark and Iceland	317	31.5 (4.3)/31.7 (4.1)	33.1 (1.3)/33.0 (1.5)	A low calorie liquid formula diet of 880 kcal/day	IVF with no previous interventions	Live birth (spontaneous IVF) Undesirable effects (miscarriage, ectopic pregnancy) Pregnancy rate (clinical, multiple) BMI change Weight change	12	Sahlgrenska University Hospital (ALFGBG-70 940), Merck AB, Solna, Sweden (an affiliate of Merck KGaA, Darmstadt, Germany), Impolin AB, Hjalmar Svensson Foundation and Jane and Dan Olsson Foundation
Espinós et al. (30), 2017 Fertility Unit of Hospital de la Santa Creu i Sant Pau-Fundacio Puigvert, Barcelona Spain	41	32.0 (3.2)/32.9 (3.9)	34.6 (3.0)/34.0 (4.1)	Diet and exercise	IVF/ICSI with no previous interventions	Live birth (IVF, cumulative) Undesirable effects (miscarriage) Pregnancy rate (clinical, multiple) Weight change Implantation rate Fertilization rate	12	Grant from the Instituto de Salud Carlos III (PI11/02816)
Moran et al. (31), 2016 Repromed, Adelaide Australia	46	33.8 (3.5)/32.5 (3.3)	34.0 (4.5)/33.9 (4.4)	A nutritionally adequate reduced energy diet and exercise intervention andcontact with investigators	A standard advice on appropriate diet and lifestyle factors influencing fertility provided face-to face at one session with no active follow-up	Live birth Undesirable effects (miscarriage) Pregnancy rate BMI change Weight change	Not reported	NHMRC Program Grant to RJN, a Brailsford Robertson Grant and The University of Adelaide in Adelaide, Australia, and sponsored with a product (Optifast VLCD) by Novartis USA
Mutsaerts et al. (32), 2016 University medical centres and general hospitals Netherlands	577	29.7 (4.5)/29.8 (4.6)	27.7 (range 24.4-31.0)/	Motivational counselling: outpatient visits, telephone consultations, assistance of an online diet diary, advise to engage in moderate intensity physical activity	Prompt infertility treatment with no previous interventions	Live birth Undesirable effects (miscarriage) Pregnancy rate (clinical, multiple) BMI change Weight change	24	Grant (50-50110-96-518) from the Netherlands Organization for Health Research and Development
Palomba et al. (33), 2010* Setting Units of Reproductive Medicine and Surgery Italy	96	28.43 (8.31)/26.50 (4.26)	31.05 (2.98)/32.3 (3.73)	Structured exercise training plus hypocaloric diet for 6 weeks, with one cycle of CC after the first 2 weeks	2 weeks of observation followed by one cycle of CC therapy	BMI change Weight change Ovulation rate Reproductive outcomes Changes in anthropometric and hormonal and metabolic parameters Compliance with the interventions	Not reported	Not reported
Rothberg et al. (34), 2016 University of Michigan (UM) Health System, Ann Arbor, Michigan USA	14	33 (5.0)/30 (4.0)	41 (4)/41 (4)	Intensive weight loss interventions consisted of 12 weeks of very-low-energy diet (800 kcal/day) plus 4 weeks of a low-calorie conventional food-based diet	Standard-of-care nutritional counselling consisted of 16 weeks of conventional food-based diet	Live birth Pregnancy rate BMI change Weight change	12	Grant from the Michigan Institute for Clinical Research (grant U040012 PI to A.R.); the core services of the Michigan Nutrition Obesity Research Centre (grant DK089503); and the Michigan Centre for Diabetes Research (grant P30DK020572)
Sim et al. (16), 2014, Royal Prince Alfred Hospital (RPAH) Fertility Unit, Sydney, Australia	49	32,9 (3.3)/32,8 (3.1)	35.1 (3.8)/38.0 (5.2)	A very-low-energy diet for the initial 6 weeks followed by a hypocaloric diet, combined with a weekly group multidisciplinary programme	Recommendations for weight loss and the same printed material as the intervention.	Live birth Undesirable effects (miscarriage) Pregnancy rate (clinical, assisted, natural) BMI change Weight change	12	National Health and Medical Research Council of Australia and from the Sydney University Nutrition Research Foundation to KAS. Prima Health Solutions provided the VLED (KicStart)


SD; Standard deviation, SE; Standard error, CC; Clomiphene citrate, BMI; Body mass index, IVF; *In vitro* fertilization, ICSI; Intracytoplasmic sperm injection, *; Palomba et al. study had 3 groups, but we include only group B and C described in the Table. The group A received structured exercise training plus hypocaloric diet for 6 weeks without CC.

**Table 2:** Summary of review findings


Outcomes	Anticipated absolute effects (95% CI)		Relative effect (95% CI)	Number of participants	Quality of the evidence
	Risk with usual care	Risk with lifestyle interventions (*)			

Live birthsIVF live births	242 per 1.000	346 per 1.000(181 to 655)	RR 1.43(0.75 to 2.71)	433 (4 RCTs)	⊕⊕⊖⊖Low^1, 2^
Live births All live births	405 per 1.000	563 per 1.000(365 to 867)	RR 1.39(0.90 to 2.14)	1034 (7 RCTs)	⊕⊕⊖⊖Low^2, 3^
All pregnancies	502 per 1.000	718 per 1.000(507 to 1.000)	RR 1.43(1.01 to 2.02)	1034 (7 RCTs)	⊕⊕⊕⊖Moderate^3^
Miscarriage	142 per 1.000	213 per 1.000(148 to 307)	RR 1.50(1.04 to 2.16)	543 (6 RCTs)	⊕⊕⊕⊖Moderate^4^


*; The risk in the intervention group [and its 95% confidence interval (CI)] is based on the assumed risk in the comparison group and the relative effect of the intervention (and its 95% CI), 1 ; Two studies had high risk of performance bias (open trials), and additional one high risk of attrition bias, 2 ; The confidence interval of effect estimate includes both an effect for the intervention and the control condition, 3 ; Five studies had high risk of performance bias or detection bias (open trials), and two reported selectively their outcomes, and 4 ; Four studies had high risk of performance bias or detection bias (open trials), three had high risk of attrition bias and one reported selectively its outcomes. Grade working group grades of evidence: High quality; Further research is very unlikely to change our confidence in the estimate of effect. Moderate quality; Further research is likely to have an important impact on our confidence in the estimate of effect and may change the estimate. Low quality; Further research is very likely to have an important impact on our confidence in the estimate of effect and is likely to change the estimate. Very low quality; We are very uncertain about the estimate.

### Effect of lifestyle interventions in primary outcomes

**Fig 3:**
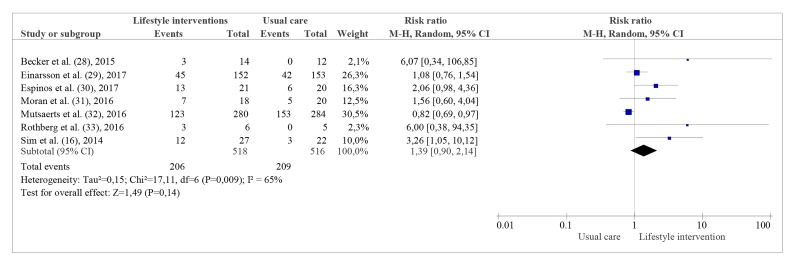
Live Birth-pooled analysis.

Seven studies reported the live birth with a total number of 1034 patients (28-34), and showed that lifestyle interventions had no effect on live birth rates (RR: 1.39, CI: 95% 0.90 to 2.14; I^2^=65%; Fig.3).

**Fig 4:**
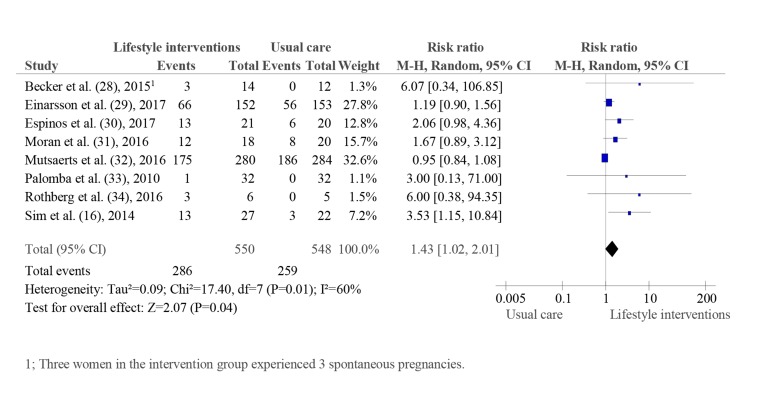
Pregnancy rate-pooled analysis.

We rated this outcome as low-quality due to limitations in study designs and imprecision in the effect estimate. On the other hand, the intervention led to higher pregnancy rates according the pooled results of seven trials including 1098 women (RR: 1.43, CI: 95% 1.02 to 2.01; I^2^=60%; Fig.4) (16, 28-31, 33). Twenty-one more women out 100 participating in a lifestyle intervention became pregnant in comparison to women receiving usual care (CI: 95% 0.5 to 38 more). 

A subgroup analysis of studies assessing interventions based on dietary restriction (28, 29) or in combination with physical activity (16, 30-32, 34) did not show changes any the effect estimates magnitude or direction (in the Materials S4, See Supplementary Online Information at www.ijfs.ir).

**Fig 5:**
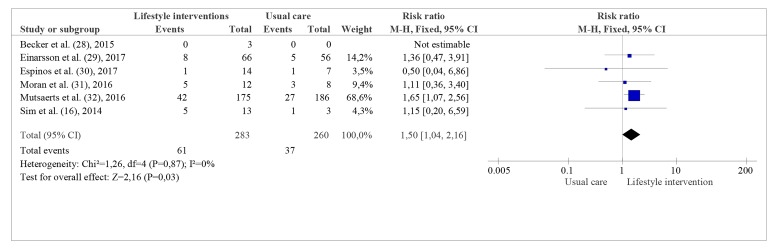
Miscarriage-pooled analysis.

Notably, the results from six studies with a total number of 543 participants (16, 28-32) showed a statistically significant increase in the risk of miscarriage in women allocated to lifestyle interventions (RR: 1.50, CI: 95% 1.04 to 2.16; I^2^=0; Fig.5), resulting in seven women more out of 100 allocated to lifestyle interventions having a miscarriage in comparison to women receiving usual care (CI: 95% 0.6 to 9 more). We rated pregnancy rates and miscarriage as moderate quality due to limitations in studies design. This increase in the risk of miscarriage disappeared in a subgroup analysis of interventions that were exclusively based on a dietary restriction according the pooled results from two trials (125 participants; RR: 1.36; 95% CI: 0.47 to 3.91; I^2^=0) (Materials S4, See Supplementary Online Information at www.ijfs.ir).

After exploring possible sources of heterogeneity, we performed a sensitivity analysis excluding from the pooled analyses one trial that could have an impact on the consistency of effect estimates (32). The results of these analyses resulted in a statistically significant increase in live birth rates that favoured the intervention (6 trials, 470 participants; RR: 1.69; 95% CI: 1.05 to 2.70; I^2^=34%), while the impact on miscarriage switched to a non-significant difference (5 trials, 182 participants; RR: 1.16; 95% CI: 0.59 to 2.30; I^2^=0%) (Materials S5) (See Supplementary Online Information at www.ijfs.ir).

### Cumulative pregnancy rate was not reported in the included studies.

Effect of lifestyle interventions in secondary outcomes The participation in a lifestyle intervention did not show differences, compared to the usual care, in the rate of ongoing pregnancies (32) (317 participants; RR: 0.91, CI: 95% 0.79 to 1.05) or implantation rates (30) (65 participants; RR: 1.32, CI: 95% 0.72 to 1.69). We rated these outcomes as low due to the imprecision in effect estimates. 

## Discussion

We included eight trials, providing a total of 1175 infertile obese women randomised to receive a type of diet and/or exercise structured program versus usual care before undergoing an assisted reproduction program. In all included studies, experimental interventions significantly lowered the women’s weight; however, there were some variations in the measure effects between the studies. The main findings of our systematic review suggests that lifestyle interventions may have little or no impact on the live birth rates of obese infertile women who wish pregnancy.

On the other hand, our results showed an increase in the risk of miscarriage rate in seven more pregnant women out of 100 receiving the intervention instead of the usual care. The sub-group analyses according to the components of the intervention of interest (dietary restriction alone or in combination with physical activity) did not have major impact on our findings. No studies reported the cumulative pregnancy rate.

Our review surveyed rigorous methodological standards, and we set the methods used in our review in a protocol prospectively registered. Most of the review steps were conducted independently by pairs of reviews to ensure the accuracy of judgements and data. We made an effort to identify all the relevant trials eligible for our inclusion criteria and asked missing data in published reports to the authors to avoid selective reporting bias. The review has also some limitations, and we obtained few missing data from trials and the data extracted from trial reports. This fact did not allow us to undertake reliable analysis to explore the effects of the intervention in terms of different characteristics of women participating in other studies, the interventions assessed or the control conditions. Also, we limited inclusion to randomised trials that allowed us to obtain reliable effect estimates but omitted the results from a body of controlled observational studies (see excluded studies at Materials S2, See Supplementary Online Information at www.ijfs. ir) that could bring light to the findings of our review. We also found some high heterogeneity related with the different types of interventions for reducing weight and the discrepancies in women’s characteristics, such as age and the baseline values of women’s weight between studies. We rated the quality of evidence for primary outcomes as moderate-to-low due to the limitations in the included studies design and the imprecision in effect estimates.

The increase in the miscarriage rate is an unexpected finding since obesity has been related to a lower oocyte quality and endometrial receptivity increasing the risk of pregnancy loss. However, the study by Mutsaerts et al. (32) in comparison with the other studies introduced clinical heterogeneity because women had lower BMI and the control group received a higher number of infertility treatments; furthermore, the assessed intervention lasted for a longer period and the study presented attrition bias (22% of losses). For these reasons, we excluded Mutsaerts et al. (32) study in the sensitivity analysis. In consequence, results changed to lifestyle interventions increased of live birth and there was not difference in the risk of miscarriage compared with the control group. These results are more consistent with recent data that show an association of weight gain ≥5% with a higher risk of pregnancy loss compared with maintaining a constant weight. The weight loss ≥5% did not associate with the increased risk of pregnancy loss (35). Other systematic reviews have reported the effect of diet and/or exercise on obese fertile women. One review (36) assessed the effect of low carbohydrate diet on fertility hormones and pregnancy in overweight and obese women with a methodology that differed from our review and with inconclusive results regarding the impact of intervention on the pregnancy rate. Another review also focused on assessed weight-loss interventions in overweight and obese women with broader inclusion criteria (the review included non-randomized studies and also assessed weight reduction drugs) (18). Pooled analysis from randomized trials showed similar results for the pregnancy rate and live birth, but did not show any increase in the rate of miscarriage, as shown by our findings.

Lifestyle intervention programmes targeted to people with overweight or obesity usually result in poor compliance rates and gender have been identified as one of the critical predictors for adherence, which is lower in women (37). On the other hand, a great majority of obese women facing an infertility treatment with interest in a supervised medical weight-loss programme would not be willing to delay the fertility treatment more than three months to attempt weight-loss (38). These considerations are relevant in the light of the review findings when making a decision to initiate a programme such those described but facing low expectations from it in terms of the fertility treatment success. In that context, an individualized and shared decision should be made exploring patient motivation and other compliance predictors, such as age, baseline BMI, and mood (37).

## Conclusion

Lifestyle interventions in obese infertile women based on dietary restrictions and physical activity probably lead to a slightly increase in the pregnancy rate compared with the usual care and make little difference in the improvement of live birth. Furthermore, our findings suggested a link between these interventions and a slightly increase of the risk of miscarriage. More research is needed in obese women undergoing infertility programs to further confirm or refute our findings. 

## Supplementary PDF


