# Supplementary material for: The Effect of Lifestyle Intervention on Pregnancy and Birth Outcomes on Obese Infertile Women: A Systematic Review and Meta-Analysis
Source: Int J Fertil Steril. 2020 Feb 25;14(1):1–9. doi: 10.22074/ijfs.2020.5921 (PMC7139226; doi:10.22074/ijfs.2020.5921)
Supplement: Supplementary file 1 [file Int-J-Fertil-Steril-14-1-s01.pdf]

Int J Fertil Steril
International Journal of Fertility & Sterility
ISSN:2008-076X eISSN:2008-0778

www.ijfs.ir

Supplementary Information for

The Effect of Lifestyle Intervention on Pregnancy and Birth Outcomes on Obese Infertile Women: A Systematic Review and Meta-Analysis

Juan J Espinós, M.D.[1*], Ivan Solà, M.D., Ph.D.[2, 3, 4], Claudia Valli, M.Sc.[2], Ana Polo, M.D.[5], Lucja Ziolkowska, M.D.[2, 6], M José Martínez-Zapata, M.D.[2, 3, 4]

1. Department of Obstetrics and Gynaecology, Hospital de la Santa Creu i Sant Pau, Barcelona, Universitat Autònoma de Barcelona (UAB), Bellaterra, Spain
2. IbCC Iberoamerican Cochrane Center, Barcelona, Spain
3. CIBERESP CIBER Epidemiología y Salud Pública (CIBERESP), Barcelona, Spain
4. IIB Sant Pau Biomedical Research Institute Sant Pau (IIB Sant Pau), Barcelona, Spain
5. Department of Reproduction Puigvert Foundation, Barcelona, Spain
6. Medical University of Silesia, Katowice, Poland

S1: Search strategies for the electronic databases.

Cochrane Central Register of Controlled Trials (CENTRAL) (The Cochrane Library) (Issue 2 of 12, February 2018)
#1 MeSH descriptor: [Infertility, Female] explode all trees and with qualifier(s): [Therapy - TH] 517
#2 MeSH descriptor: [Reproductive Techniques, Assisted] explode all trees 3380
#3 MeSH descriptor: [Fertilization in Vitro] explode all trees 2176
#4 infertile*:ti 561
#5 fertile*:ti 66
#6 ivf:ti,ab 3675
#7 (in next vitro next fertilisation):ti,ab 292
#8 (in next vitro next fertilization):ti,ab 4304
#9 #1 or #2 or #3 or #4 or #5 or #6 or #7 or #8 6264
#10 MeSH descriptor: [Obesity] explode all trees 10432
#11 MeSH descriptor: [Overweight] explode all trees 11516
#12 MeSH descriptor: [Body Mass Index] explode all trees 8616
#13 overweight*:ti,ab 8890
#14 obes*:ti,ab 21154
#15 #10 or #11 or #12 or #13 or #14 28524
#16 MeSH descriptor: [Life Style] explode all trees 4181
#17 MeSH descriptor: [Diet, Reducing] explode all trees 1935
#18 MeSH descriptor: [Caloric Restriction] explode all trees 567
#19 MeSH descriptor: [Exercise] explode all trees 19396
#20 MeSH descriptor: [Exercise Therapy] explode all trees 10763
#21 MeSH descriptor: [Physical Fitness] explode all trees 2711
#22 MeSH descriptor: [Weight Loss] explode all trees 4783
#23 lifestyle:ti,ab 8425
#24 (life next style):ti,ab 593
#25 diet:ti,ab 24638
#26 (weight next loss):ti,ab 10744
#27 (caloric next restricti*):ti,ab 437
#28 (physical next activity):ti,ab 13170
#29 exercise:ti,ab 47236
#30 #16 or #17 or #18 or #19 or #20 or #21 or #22 or #23 or #24 or #25 or #26 or #27 or #28 or #29 89104
#31 #9 and #15 and #30 39

MEDLINE (via PubMed)
#1 "Infertility, Female/therapy"[Mesh] 12154
#2 "Reproductive Techniques, Assisted"[Majr] 42279
#3 "Fertilization in Vitro"[Mesh] 32425
#4 infertile*[ti] 5039

*Corresponding Address: Department of Obstetrics and Gynecology, "Filippo Del Ponte" Hospital, University of Insubria, Piazza Biroldi 1, 21100, Varese, Italy
Email: antoniosimone.lagana@uninsubria.it

#5 fertile*[ti] 2141
#6 ivf[tiab] 21090
#7 in vitro fertilisation[tiab] 1703
#8 in vitro fertilization[tiab] 19424
#9 #1 OR #2 OR #3 OR #4 OR #5 OR #6 OR #7 OR #8 72866
#10 "Obesity"[Mesh] 182422
#11 "Overweight"[Mesh] 187345
#12 "Body Mass Index"[Mesh] 107729
#13 overweight*[tiab] 56755
#14 obes*[tiab] 253456
#15 #10 OR #11 OR #12 OR #13 OR #14 360337
#16 "Life Style"[Mesh] 79841
#17 "Diet, Reducing"[Mesh] 10608
#18 "Caloric Restriction"[Mesh] 5129
#19 "Exercise"[Mesh] 161938
#20 "Exercise Therapy"[Mesh] 41658
#21 "Physical Fitness"[Mesh] 25682
#22 "Weight Loss"[Mesh] 36095
#23 "diet therapy"[sh] 45985
#24 lifestyle[tiab] 72996
#25 life style[tiab] 10116
#26 diet[tiab] 284169
#27 weight loss[tiab] 73734
#28 caloric restricti*[tiab] 3992
#29 physical activity[tiab] 86806
#30 exercise[tiab] 228914
#31 #16 OR #17 OR #18 OR #19 OR #20 OR #21 OR #22 OR #23 OR #24 OR #25 OR #26 OR #27 OR #28 OR #29 OR #30 833989
#32 #9 AND #15 AND #31 264

EMBASE (via Ovid Embase 1980 to 2018 Week 11)

1 exp female infertility/ (40911)
2 exp female subfertility/ (849)
3 exp reproductive procedure/ (1017)
4 exp in vitro fertilization/ (59002)
5 exp fertilization in vitro/ (59002)
6 infertile*.ti. (6939)
7 fertile*.ti. (2349)
8 IVF.ti,ab. (34880)
9 in vitro fertilisation.ti,ab. (2438)
10 in vitro fertilization.ti,ab. (24178)
11 1 or 2 or 3 or 4 or 5 or 6 or 7 or 8 or 9 or 10 (117865)
12 exp lifestyle/ (107893)
13 exp lifestyle modification/ (30765)
14 exp behavior modification/ (7373)
15 *health program/ (18683)
16 diet/ (193597)
17 low calory diet/ (8467)
18 caloric restriction/ (13309)
19 exercise/ (237755)
20 physical activity/ (123946)
21 weight reduction/ (147595)
22 weight change/ (11442)
23 lifestyle.ti,ab. (99541)
24 life style.ti,ab. (14595)
25 diet.ti,ab. (345760)
26 weight loss.ti,ab. (112914)
27 caloric restricti*.ti,ab. (5003)
28 physical activity.ti,ab. (113778)
29 exercise.ti,ab. (285644)
30 12 or 13 or 14 or 15 or 16 or 17 or 18 or 19 or 20 or 21 or 22 or 23 or 24 or 25 or 26 or 27 or 28 or 29 (1139166)
31 exp body mass/ (323646)
32 obesity/ (353729)
33 overweight*.ti,ab. (83203)
34 obes*.ti,ab. (357374)
35 31 or 32 or 33 or 34 (664028)
36 11 and 30 and 35 (1217)
37 random:.tw. or clinical trial:.mp. or exp health care quality/ (4455876)
38 36 and 37 (423)

S2: Excluded studies

Reasons for exclusion

Study ID	Reason for exclusion
Clark et al. (1), 1995, Crosignani et al. (2), 2003, Evenson et al. (3), 2014, Galletly et al. (4), 1996, Hollmann et al. (5), 1996, Karayiannis et al. (6), 2018, Khaskheli et al. (7), 2013, Kort et al. (8), 2014, Mahoney (9), 2013, Marsh et al. (10), 2010, Matalliotakis et al. (11), 2008, Miller et al. (12), 2008, Miranda-Furtado et al. (13), 2016, Morris et al. (14), 2006, Palomba et al. (15), 2014, Parent et al. (16), 2016, Pasquali et al. (17), 2010, Ramezanzadeh et al. (18), 2012, Tsagareli et al. (19), 2006	Cohort study
Maheshwari et al. (20), 2010, Ryu et al. (21), 2017, Panidis and Farmakiotis (22), 2000, Duval et al. (23), 2015, Laredo (24), 2006, Oyesanya et al. (25), 2009, Cohen et al. (26), 2011, Pinborg et al. (27), 2013, Sacha et al. (28), 2018	Ineligible study design
van Dammen et al. (29), 2018, Moran et al. (30), 2011, Mutsaerts et al. (31), 2010, Palomba et al. (32), 2008, van Oers et al. (33), 2017, van Oers et al. (34), 2017	Secondary publications from included studies
Guzick et al. (35), 1994, Hernández García et al. (36), 1999, Sweatt et al. (37), 2015	Did not include eligible outcomes
Legro et al. (38), 2015	Ineligible intervention (the lifestyle intervention included the use of oral contraceptive pills

S3: Pooled analyses for main outcomes

S3.1: Live birth

Study or subgroup	Lifestyle interventions Events	Lifestyle interventions Total	Usual care Events	Usual care Total	Weight	Risk ratio M-H, Random, 95% CI
Becker et al. (39), 2015	3	14	0	12	2,1%	6,07 [0,34, 106,85]
Einarsson et al. (40), 2017	45	152	42	153	26,3%	1,08 [0,76, 1,54]
Espinos et al. (41), 2017	13	21	6	20	16,3%	2,06 [0,98, 4,36]
Moran et al. (42), 2016	7	18	5	20	12,5%	1,56 [0,60, 4,04]
Mutsaerts et al. (43), 2016	123	280	153	284	30,5%	0,82 [0,69, 0,97]
Rothberg et al. (44), 2016	3	6	0	5	2,3%	6,00 [0,38, 94,35]
Sim et al. (45), 2014	12	27	3	22	10,0%	3,26 [1,05, 10,12]
Subtotal (95% CI)		518		516	100,0%	1,39 [0,90, 2,14]
Total events	206		209			

Heterogeneity: Tau² = 0,15; Chi² = 17,11, df = 6 (P = 0,009); I² = 65%
Test for overall effect: Z=1,49 (P = 0,14)

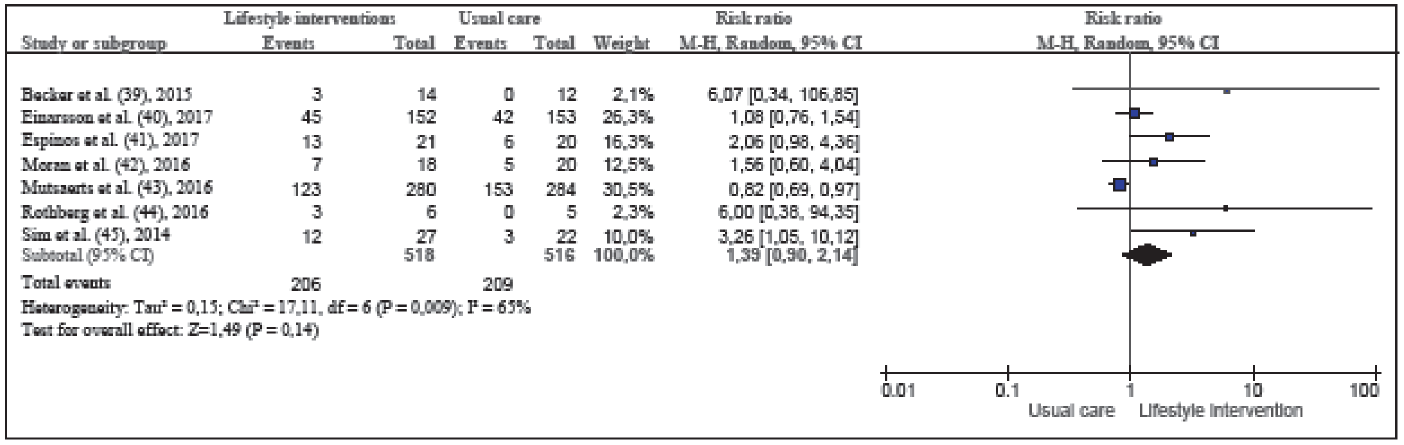

S3.2: Pregnancy

Study or subgroup	Lifestyle interventions Events	Lifestyle interventions Total	Usual care Events	Usual care Total	Weight	Risk ratio M-H, Random, 95% CI
Becker et al. (39), 2015	3	14	0	12	2,1%	6,07 [0,34, 106,85]
Einarsson et al. (40), 2017	45	152	42	153	26,3%	1,08 [0,76, 1,54]
Espinos et al. (41), 2017	13	21	6	20	16,3%	2,06 [0,98, 4,36]
Moran et al. (42), 2016	7	18	5	20	12,5%	1,56 [0,60, 4,04]
Mutsaerts et al. (43), 2016	123	280	153	284	30,5%	0,82 [0,69, 0,97]
Rothberg et al. (44), 2016	3	6	0	5	2,3%	6,00 [0,38, 94,35]
Sim et al. (45), 2014	12	27	3	22	10,0%	3,26 [1,05, 10,12]
Subtotal (95% CI)		518		516	100,0%	1,39 [0,90, 2,14]
Total events	206		209			

Heterogeneity: Tau² = 0,15; Chi² = 17,11, df = 6 (P = 0,009); I² = 65%
Test for overall effect: Z=1,49 (P = 0,14)

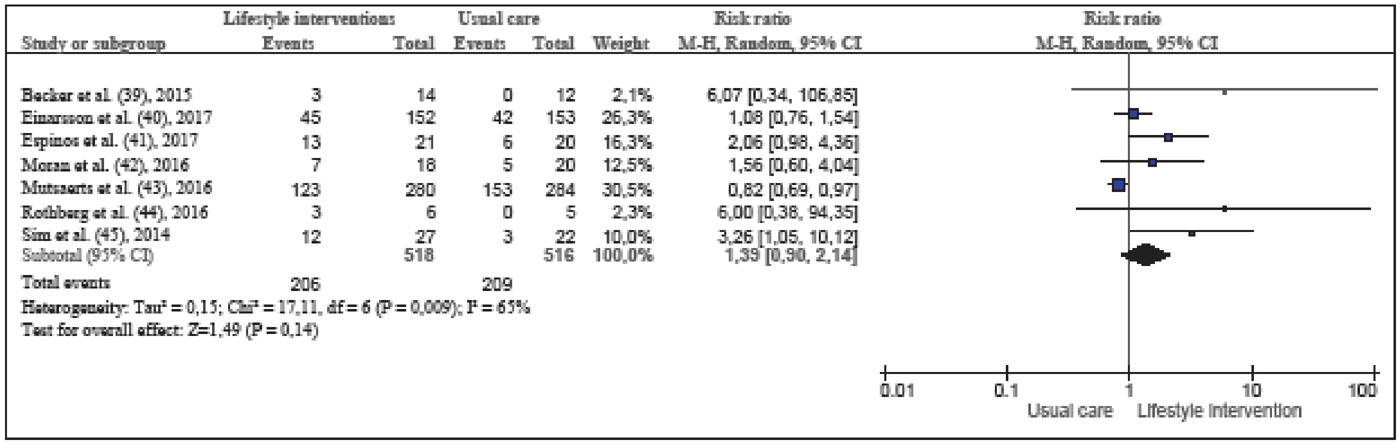

S3.3: Miscarriage

Study or Subgroup	Lifestyle interventions Events	Lifestyle interventions Total	Usual care Events	Usual care Total	Weight	Risk ratio M-H, Fixed, 95% CI
Becker et al. (39), 2015	0	3	0	0		Not estimable
Einarsson et al. (40), 2017	8	66	5	56	14,2%	1,36 [0,47, 3,91]
Espinos et al. (41), 2017	1	14	1	7	3,5%	0,50 [0,04, 6,86]
Moran et al. (42), 2016	5	12	3	8	9,4%	1,11 [0,36, 3,40]
Mutsaerts et al. (43), 2016	42	175	27	186	68,6%	1,65 [1,07, 2,56]
Sim et al. (45),2014	5	13	1	3	4,3%	1,15 [0,20, 6,59]
Total (95% CI)		283		260	100,0%	1,50 [1,04, 2,16]
Total events	61		37			

Heterogeneity: Chi²=1,26, df = 4 (P=0,87); I²=0%
Test for overall effect: Z=2,16 (P=0,03)

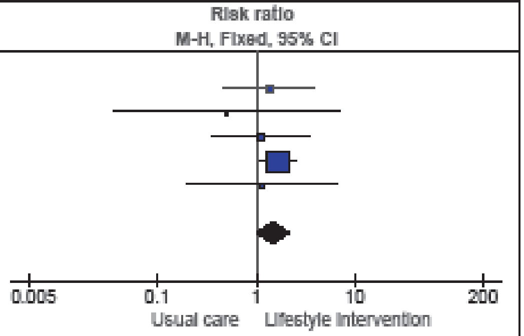

S4: Pooled sub group analyses according to the interventions components (dietary intake alone or combined with physical acivity)

S4.1: Live birth: diet versus usual care

	Usual diet		Control			Risk Ratio
Study or Subgroup	Events	Total	Events	Total	Weight	M-H, Fixed, 95% CI
**5.1.3 All live birth**						
Becker 2015	3	14	0	12	1,3%	6,07 [0,34, 106,95]
Einarsson 2017	45	152	42	153	98,7%	1,08 [0,76, 1,54]
**Subtotal (95% CI)**		**166**		**165**	**100,0%**	**1,14 [0,80, 1,62]**
Total events	48		42			
Heterogeneity: Chi² = 1,40, df = 1 (P = 0,24); I² = 29%						
Test for overall effect: Z = 0,74 (P = 0,46)						
Test for subgroup differences: Not applicable						

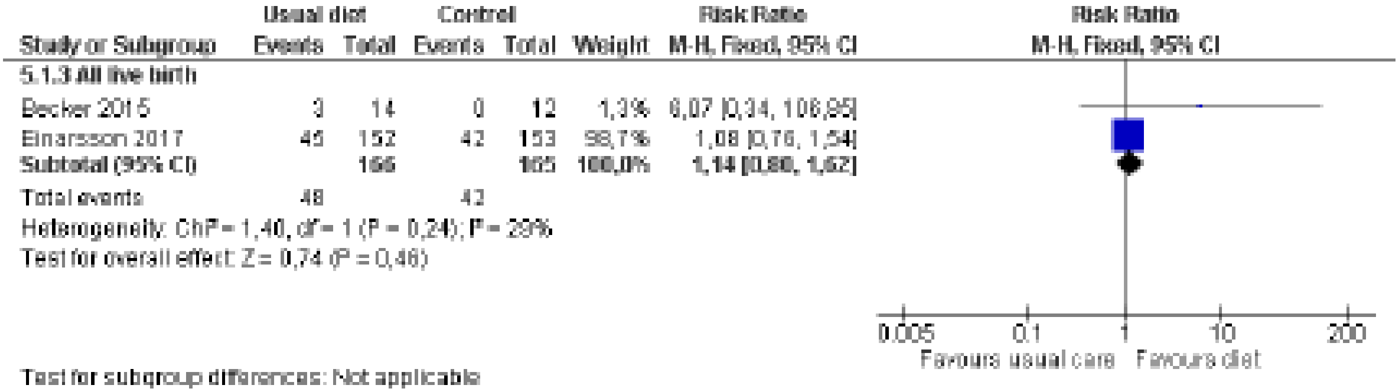

S4.2: Live birth: diet and physical activity versus usual care

	Exercise plus diet		Control			Risk Ratio
Study or Subgroup	Events	Total	Events	Total	Weight	M-H, Random, 95% CI
**5.2.3 All live birth**						
Espinos 2017	13	21	6	20	24,0%	2,06 [0,98, 4,36]
Moran 2016	7	18	5	20	20,5%	1,56 [0,60, 4,04]
Mutsaerts 2016	123	280	153	284	32,4%	0,82 [0,69, 0,97]
Rothberg 2016	3	6	0	5	5,4%	6,00 [0,38, 94,35]
Sim 2014	12	27	3	22	17,7%	3,26 [1,05, 10,12]
**Subtotal (95% CI)**		**352**		**351**	**100,0%**	**1,66 [0,82, 3,33]**
Total events	158		167			
Heterogeneity: Tau² = 0,38; Chi² = 14,46, df = 4 (P = 0,006); I² = 72%						
Test for overall effect: Z = 1,41 (P = 0,16)						
Test for subgroup differences: Not applicable						

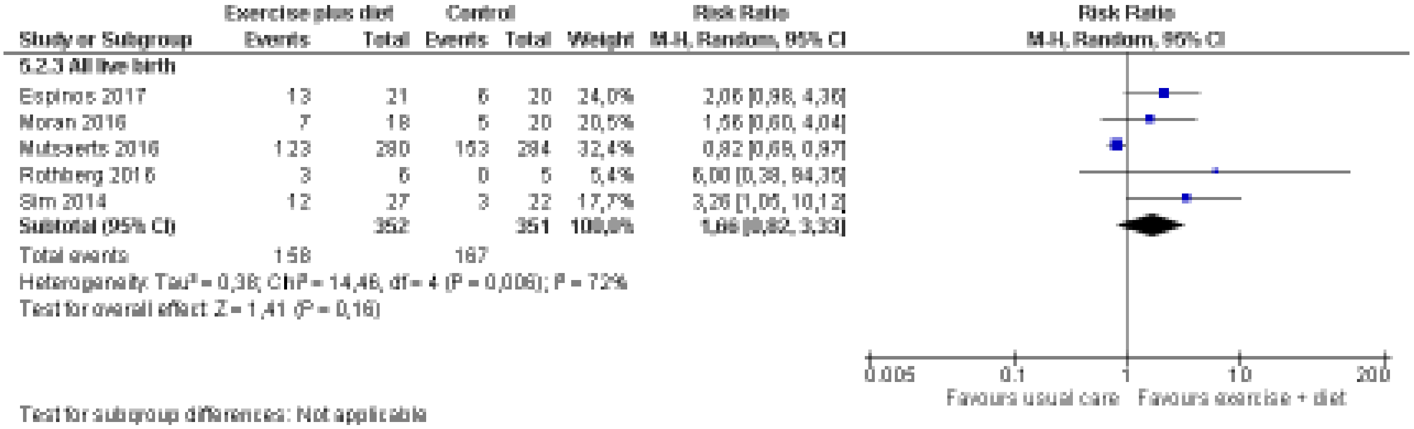

S4.3: Miscarriage: diet versus usual care

	Diet		Usual care			Risk Ratio
Study or Subgroup	Events	Total	Events	Total	Weight	M-H, Fixed, 95% CI
Becker 2015	0	3	0	0		Not estimable
Einarsson 2017	8	66	5	56	100,0%	1,36 [0,47, 3,91]
**Total (95% CI)**		**69**		**56**	**100,0%**	**1,36 [0,47, 3,91]**
Total events	8		5			
Heterogeneity: Not applicable						
Test for overall effect: Z = 0,57 (P = 0,57)						

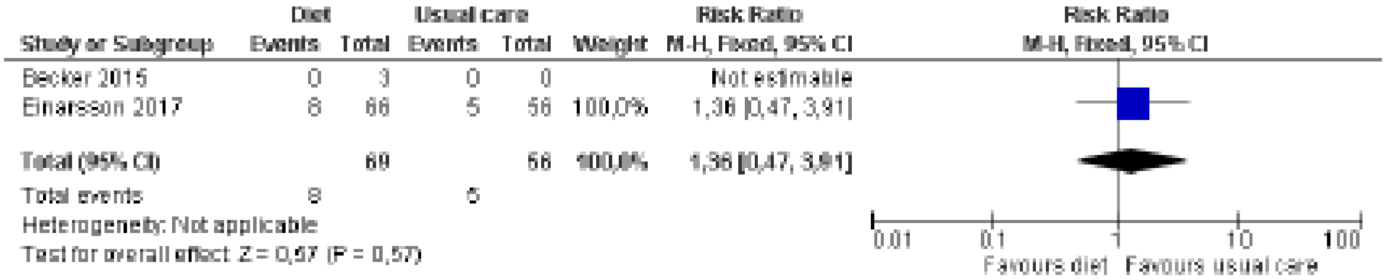

S4.4: Miscarriage: diet and physical activity versus usual care

Study or Subgroup	Exercise plus diet Events	Exercise plus diet Total	Usual care Events	Usual care Total	Weight	Risk Ratio M-H, Fixed, 95% CI
Espinos 2017	1	14	1	7	4,1%	0,50 [0,04, 6,86]
Moran 2016	5	12	3	8	11,0%	1,11 [0,36, 3,40]
Mutsaerts 2016	42	175	27	186	80,0%	1,65 [1,07, 2,56]
Sim 2014	5	13	1	3	5,0%	1,15 [0,20, 6,58]
**Total (95% CI)**		**214**		**204**	**100,0%**	**1,52 [1,03, 2,25]**
Total events	53		32			

Heterogeneity: $Chi^2 = 1,23$, df = 3 (P = 0,75); $I^2 = 0\%$
Test for overall effect: Z = 2,10 (P = 0,04)

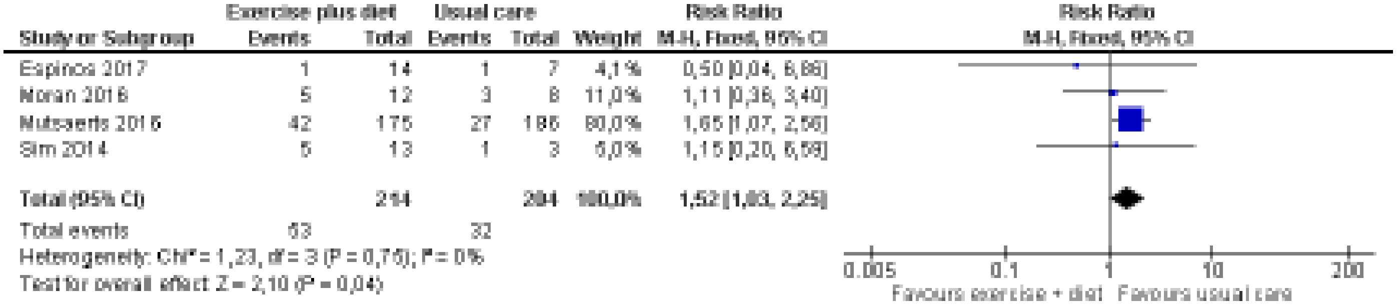

S5: Pooled sensitivity analyses after excluding Mutsaerts et al. (43), 2016
S5.1: Live birth

Study or Subgroup	Lifestyle interventions Events	Lifestyle interventions Total	Usual care Events	Usual care Total	Weight	Risk Ratio M-H, Random, 95% CI
**6.1.4 All live birth**						
Becker 2015	3	14	0	12	2,6%	6,07 [0,34, 108,85]
Einarsson 2017	45	152	42	153	41,8%	1,08 [0,76, 1,54]
Espinos 2017	13	21	6	20	22,9%	2,06 [0,98, 4,36]
Moran 2016	7	18	5	20	16,8%	1,56 [0,60, 4,04]
Rothberg 2016	3	6	0	5	2,8%	6,00 [0,38, 94,35]
Sim 2014	12	27	3	22	13,1%	3,26 [1,05, 10,13]
**Subtotal (95% CI)**		**238**		**232**	**100,0%**	**1,69 [1,05, 2,70]**
Total events	83		56			

Heterogeneity: $Tau^2 = 0,10$; $Chi^2 = 7,46$, df = 5 (P = 0,19); $I^2 = 33\%$
Test for overall effect: Z = 2,18 (P = 0,03)

Test for subgroup differences: Not applicable

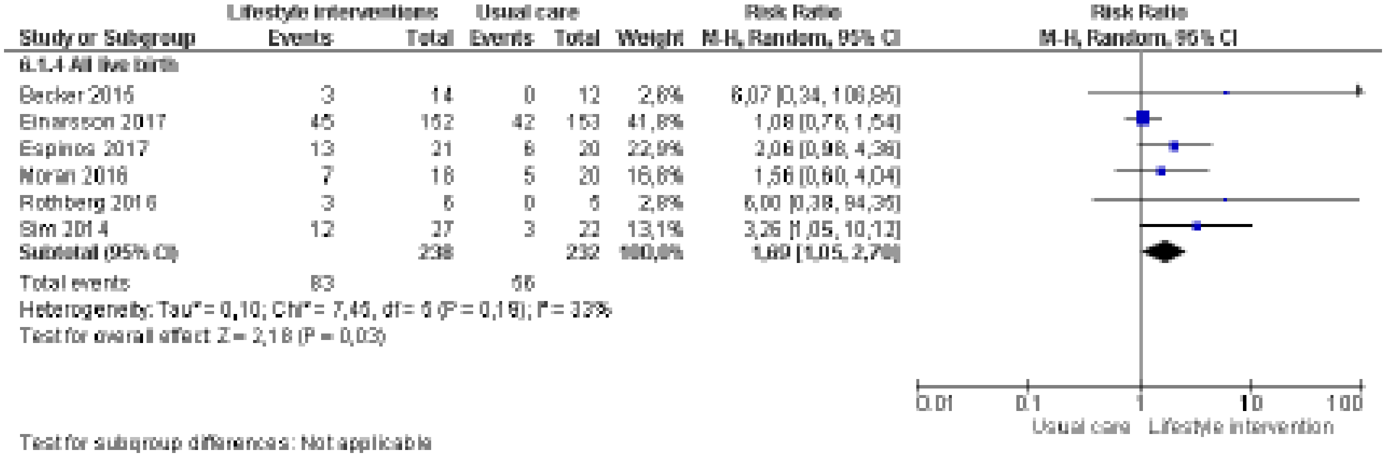

S5.2: Pregnancy rate

Study or Subgroup	Lifestyle interventions Events	Lifestyle interventions Total	Usual care Events	Usual care Total	Weight	Risk Ratio M-H, Random, 95% CI
Becker 2015 (1)	3	14	0	12	1,9%	6,07 [0,34, 108,85]
Einarsson 2017	66	152	56	153	43,2%	1,19 [0,90, 1,58]
Espinos 2017	13	21	6	20	18,8%	2,06 [0,98, 4,36]
Moran 2016	12	18	8	20	23,4%	1,67 [0,89, 3,12]
Rothberg 2016	3	6	0	5	2,1%	6,00 [0,38, 94,35]
Sim 2014	13	27	3	22	10,5%	3,53 [1,15, 10,84]
**Total (95% CI)**		**238**		**232**	**100,0%**	**1,71 [1,14, 2,56]**
Total events	110		73			

Heterogeneity: $Tau^2 = 0,08$; $Chi^2 = 7,60$, df = 5 (P = 0,18); $I^2 = 34\%$
Test for overall effect: Z = 2,59 (P = 0,010)

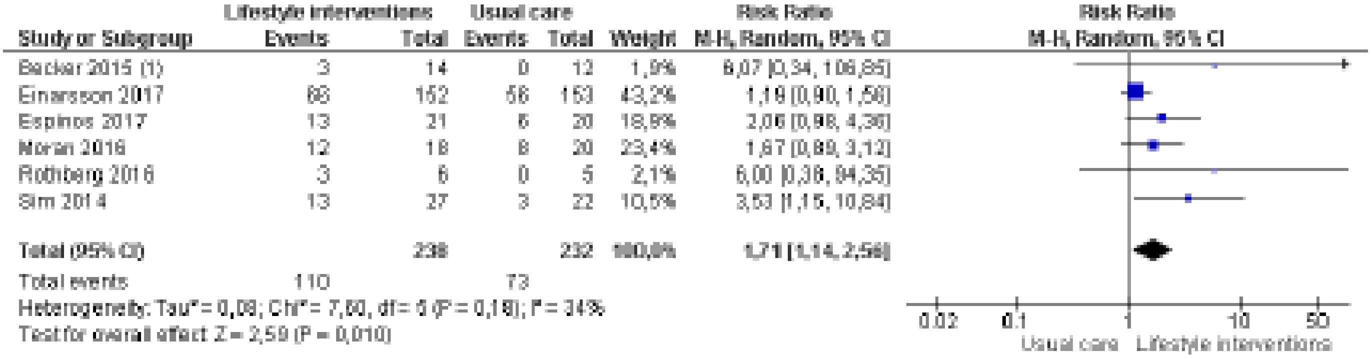

Footnotes
(1) Three women in the intervention group experienced 3 spontaneous pregnancies.

S5.3: Miscarriage

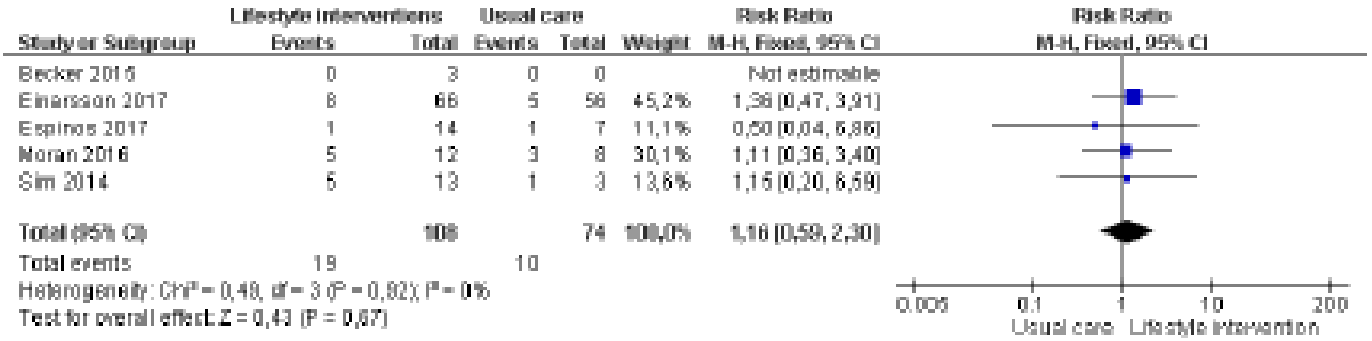

References

1. Clark AM, Ledger W, Galletly C, Tomlinson L, Blaney F, Wang X, et al. Weight loss results in significant improvement in pregnancy and ovulation rates in anovulatory obese women. Hum Reprod. 1995; 10(10): 2705-2712.
2. Crosignani PG, Colombo M, Vegetti W, Somigliana E, Gessati A, Ragni G. Overweight and obese anovulatory patients with polycystic ovaries: Parallel improvements in anthropometric indices, ovarian physiology and fertility rate induced by diet. Hum Reprod. 2003; 18(9): 1928-1932.
3. Evenson KR, Calhoun KC, Herring AH, Pritchard D, Wen F, Steiner AZ. Association of physical activity in the past year and immediately after in vitro fertilization on pregnancy. Fertil Steril. 2014; 101(4): 1047-1054. e5.
4. Galletly C, Clark A, Tomlinson L, Blaney F. Improved pregnancy rates for obese, infertile women following a group treatment program. An open pilot study. Gen Hosp Psychiatry. 1996; 18(3): 192-195.
5. Hollmann M, Runnebaum B, Gerhard I. Effects of weight loss on the hormonal profile in obese, infertile women. Hum Reprod. 1996; 11(9): 1884-1891.
6. Karayiannis D, Kontogianni MD, Mendorou C, Mastrominas M, Yiannakouris N. Adherence to the Mediterranean diet and IVF success rate among non-obese women attempting fertility. Hum Reprod. 2018; 33(3): 494-502.
7. Khaskheli MN, Baloch S, Baloch AS. Infertility and weight reduction: influence and outcome. J Coll Physicians Surg Pak. 2013; 23(10): 798-801.
8. Kort JD, Winget C, Kim SH, Lathi RB. A retrospective cohort study to evaluate the impact of meaningful weight loss on fertility outcomes in an overweight population with infertility. Fertil Steril. 2014; 101(5): 1400-1403.
9. Mahoney D. Lifestyle modification intervention among infertile overweight and obese women with polycystic ovary syndrome. J Am Assoc Nurse Pract. 2014; 26(6): 301-308.
10. Marsh KA, Steinbeck KS, Atkinson FS, Petocz P, Brand-Miller JC. Effect of a low glycemic index compared with a conventional healthy diet on polycystic ovary syndrome. Am J Clin Nutr. 2010; 92(1): 83-92.
11. Matalliotakis I, Cakmak H, Arici A, Goumenou A, Fragouli Y, Sakkas D. Epidemiological factors influencing IVF outcome: Evidence from the Yale IVF program. J Obstet Gynaecol. 2008; 28(2): 204-208.
12. Miller PB, Forstein DA, Styles S. Effect of short-term diet and exercise on hormone levels and menses in obese, infertile women. J Reprod Med. 2008; 53(5): 315-319.
13. Miranda-Furtado CL, Ramos FK, Kogure GS, Santana-Lemos BA, Ferriani RA, Calado RT, et al. A nonrandomized trial of progressive resistance training intervention in women with polycystic ovary syndrome and its implications in telomere content. Reprod Sci. 2016; 23(5): 644-654.
14. Morris SN, Missmer SA, Cramer DW, Powers RD, McShane PM, Hornstein MD. Effects of lifetime exercise on the outcome of in vitro fertilization. Obstet Gynecol. 2006; 108(4): 938-945.
15. Palomba S, Falbo A, Valli B, Morini D, Villani MT, Nicoli A, et al. Physical activity before IVF and ICSI cycles in infertile obese women: an observational cohort study. Reprod Biomed Online. 2014; 29(1): 72-79.
16. Parent C, Pigeyre M, Pleuvret A, Thomas P, Deruelle P, Dewailly D, et al. Infertility: a key time to follow a medical nutritional management. Our experience on 78 patients. Gynecol Obstet Fertil. 2016; 44(4): 218-224.
17. Pasquali R, Gambineri A, Cavazza C, Ibarra Gasparini D, Ciampaglia W, Cognigni GE, et al. Heterogeneity in the responsiveness to long-term lifestyle intervention and predictability in obese women with polycystic ovary syndrome. Eur J Endocrinol. 2011; 164(1): 53-60.
18. Ramezanzadeh F, Kazemi A, Yavari P, Nasr-Esfahani MH, Nejat S, Rahimi-Foroshani A, et al. Impact of body mass index versus physical activity and calorie intake on assisted reproduction outcomes. Eur J Obstet Gynecol Reprod Biol. 2012; 163(1): 52-56.
19. Tsagareli V, Noakes M, Norman RJ. Effect of a very-low-calorie diet on in vitro fertilization outcomes. Fertil Steril. 2006; 86(1): 227-229.
20. Maheshwari A, Scotland G, Bell J, McTavish A, Hamilton M, Bhattacharya S. The direct health services costs of providing assisted reproduction services in overweight or obese women: a retrospective cross-sectional analysis. Hum Reprod. 2009; 24(3): 633-639.
21. Ryu A, Kim TH, Park YJ, Enkhbold T. Re: self-weighing and simple dietary advice for overweight and obese pregnant women to reduce obstetric complications without impact on quality of life: a randomised controlled trial: weight control in pregnant women. BJOG. 2017; 124(4): 697-698.
22. Panidis D, Farmakiotis D. Treatment of infertility in the polycystic ovary syndrome. N Engl J Med. 2007; 356(19): 1999.
23. Duval K, Langlois MF, Carranza-Mamane B, Pesant MH, Hivert MF, Poder TG, et al. The Obesity-Fertility Protocol: a randomized controlled trial assessing clinical outcomes and costs of a transferable interdisciplinary lifestyle intervention, before and during pregnancy, in obese infertile women. BMC Obes. 2015; 2: 47.
24. Laredo SE. Obesity, polycystic ovary syndrome, infertility treatment: asking obese women to lose weight before treatment increases stigmatisation. BMJ. 2006; 332(7541): 609.
25. Oyesanya OA, van Wely M, Clarke MJ. Life-style modification, non-pharmacological and pharmacological strategies for obese subfertile women. Cochrane database of systematic reviews. 2009; (1).
26. Cohen J, Grudzinskas G, Johnson M. And now for some weighty matters. Reprod Biomed Online. 2011; 23(4): 401-402.
27. Pinborg A, Petersen GL, Schmidt L. Recent insights into the influence of female bodyweight on assisted reproductive technology outcomes. Womens Health (Lond). 2013; 9(1): 1-4.
28. Sacha CR, Page CM, Goldman RH, Ginsburg ES, Zera CA. Are women with obesity and infertility willing to attempt weight loss prior to fertility treatment? Obes Res Clin Pract. 2018; 12(1): 125-128.
29. van Dammen L, Wekker V, van Oers AM, Mutsaerts MAQ, Painter RC, Zwinderman AH, et al. Effect of a lifestyle intervention in obese infertile women on cardiometabolic health and quality of life: A randomized controlled trial. PLoS One. 2018; 13(1): e0190662.
30. Moran L, Tsagareli V, Norman R, Noakes M. Diet and IVF pilot study: short-term weight loss improves pregnancy rates in overweight/obese women undertaking IVF. Aust N Z J Obstet Gynaecol. 2011; 51(5): 455-459.
31. Mutsaerts MA, Groen H, ter Bogt NC, Bolster JH, Land JA, Bemelmans WJ, et al. The lifestyle study: costs and effects of a structured lifestyle program in overweight and obese subfertile women to re-

duce the need for fertility treatment and improve reproductive outcome. A randomised controlled trial. BMC Womens Health. 2010; 10: 22.

32. Palomba S, Giallauria F, Falbo A, Russo T, Oppedisano R, Tolino A, et al. Structured exercise training programme versus hypocaloric hyperproteic diet in obese polycystic ovary syndrome patients with anovulatory infertility: a 24-week pilot study. Hum Reprod. 2008; 23(3): 642-650.
33. van Oers AM, Groen H, Mutsaerts MA, Burggraaff JM, Kuchenbecker WK, Perquin DA, et al. Effectiveness of lifestyle intervention in subgroups of obese infertile women: a subgroup analysis of a RCT. Hum Reprod. 2017; 31(12): 2704-2713.
34. van Oers AM, Mutsaerts MAQ, Burggraaff JM, Kuchenbecker WKH, Perquin DAM, Koks CAM, et al. Cost-effectiveness analysis of lifestyle intervention in obese infertile women. Hum Reprod. 2017; 32(7): 1418-1426.
35. Guzick DS, Wing R, Smith D, Berga SL, Winters SJ. Endocrine consequences of weight loss in obese, hyperandrogenic, anovulatory women. Fertil Steril. 1994; 61(4): 598-604.
36. Hernández García IA, Gutiérrez Gutiérrez AM, Gallardo Lozano E. Effect of weight reduction on the clinical and hormonal condition of obese anovulatory women. Ginecol Obstet Mex. 1999; 67: 433-437.
37. Sweatt K, Ovalle F, Azziz R, Gower B. The effect of diet and exercise in women with polycystic ovary syndrome. FASEB J. 2015; 29(Suppl 1).
38. Legro RS, Dodson WC, Kris-Etherton PM, Kunselman AR, Stetter CM, Williams NI, et al. Randomized Controlled trial of preconception interventions in infertile women with polycystic ovary syndrome. J Clin Endocrinol Metab. 2015; 100(11): 4048-4058.
39. Becker GF, Passos EP, Moulin CC. Short-term effects of a hypocaloric diet with low glycemic index and low glycemic load on body adiposity, metabolic variables, ghrelin, leptin, and pregnancy rate in overweight and obese infertile women: a randomized controlled trial. Am J Clin Nutr. 2015; 102(6): 1365-1372.
40. Einarsson S, Bergh C, Friberg B, Pinborg A, Klajnbard A, Karlström PO, et al. Weight reduction intervention for obese infertile women prior to IVF: a randomized controlled trial. Hum Reprod. 2017; 32(8): 1621-1630.
41. Espinós JJ, Polo A, Sánchez-Hernández J, Bordas R, Pares P, Martínez O, et al. Weight decrease improves live birth rates in obese women undergoing IVF: a pilot study. Reprod Biomed Online. 2017; 35(4): 417-424.
42. Moran LJ, Tsagareli V, Noakes M, Norman R. Altered preconception fatty acid intake is associated with improved pregnancy rates in overweight and obese women undertaking in vitro fertilisation. Nutrients. 2016; 8(1). pii: E10.
43. Mutsaerts MA, van Oers AM, Groen H, Burggraaff JM, Kuchenbecker WK, Perquin DA, et al. Randomized trial of a lifestyle program in obese infertile women. N Engl J Med. 2016; 374(20): 1942-1953.
44. Rothberg A, Lanham M, Randolph J, Fowler C, Miller M, Smith Y. The Feasibility of a brief, intensive weight loss intervention to improve reproductive outcomes in obese, subfertile women: a pilot study. Fertil Steril. 2016; 106(5): 1212-1220.
45. Sim KA, Dezarnaulds GM, Denyer GS, Skilton MR, Caterson ID. Weight loss improves reproductive outcomes for obese women undergoing assisted reproductive technology: a randomised controlled trial. Clin Obes. 2014; 14: 792-805.
